# Unilateral nephrectomy diminishes ischemic acute kidney injury through enhanced perfusion and reduced pro-inflammatory and pro-fibrotic responses

**DOI:** 10.1371/journal.pone.0190009

**Published:** 2017-12-21

**Authors:** Casper Kierulf-Lassen, Per Mose Nielsen, Haiyun Qi, Mads Damgaard, Christoffer Laustsen, Michael Pedersen, Søren Krag, Henrik Birn, Rikke Nørregaard, Bente Jespersen

**Affiliations:** 1 Department of Renal Medicine, Aarhus University Hospital, Aarhus, Denmark; 2 Department of Clinical Medicine, Aarhus University, Aarhus, Denmark; 3 MR Research Centre, Aarhus University, Aarhus, Denmark; 4 Comparative Medicine Lab, Aarhus University, Aarhus, Denmark; 5 Department of Pathology, Aarhus University Hospital, Aarhus, Denmark; 6 Department of Biomedicine, Aarhus University, Aarhus, Denmark; University Medical Center Utrecht, NETHERLANDS

## Abstract

While unilateral nephrectomy (UNx) is suggested to protect against ischemia-reperfusion injury (IRI) in the remaining kidney, the mechanisms underlying this protection remain to be elucidated. In this study, functional MRI was employed in a renal IRI rat model to reveal global and regional changes in renal filtration, perfusion, oxygenation and sodium handling, and microarray and pathway analyses were conducted to identify protective molecular mechanisms. Wistar rats were randomized to either UNx or sham UNx immediately prior to 37 minutes of unilateral renal artery clamping or sham operation under sevoflurane anesthesia. MRI was performed 24 hours after reperfusion. Blood and renal tissue were harvested. RNA was isolated for microarray analysis and QPCR validation of gene expression results. The perfusion (T_1_ value) was significantly enhanced in the medulla of the post-ischemic kidney following UNx. UNx decreased the expression of fibrogenic genes, i.a. *Col1a1*, *Fn1* and *Tgfb1* in the post-ischemic kidney. This was associated with a marked decrease in markers of activated myofibroblasts (*Acta2*/*α-Sma* and *Cdh11*) and macrophages (*Ccr2*). This was most likely facilitated by down-regulation of *Pdgfra*, thus inhibiting pericyte-myofibroblast differentiation, chemokine production (*Ccl2*/*Mcp1*) and macrophage infiltration. UNx reduced ischemic histopathologic injury. UNx may exert renoprotective effects against IRI through increased perfusion in the renal medulla and alleviation of the acute pro-inflammatory and pro-fibrotic responses possibly through decreased myofibroblast activation. The identified pathways involved may serve as potential therapeutic targets and should be taken into account in experimental models of IRI.

## Introduction

Renal hypoperfusion and ischemia-reperfusion injury (IRI) are leading causes of acute kidney injury [1]. Ischemic tolerance is enhanced, when unilateral nephrectomy (UNx) is performed prior to ischemia, creating a preconditioning stimulus, which diminishes both functional impairment and structural injury [2,3]. The elicited recovery stimulus is independent of compensatory renal growth, since it can be generated by UNx performed immediately prior to sustained ischemia [3]. Few published studies have addressed the mechanisms underlying the renoprotective effects, although such information may provide valuable insights into fundamental IRI mechanisms and novel repair/regenerative mechanisms.

UNx triggers both acute hemodynamic and neurohormonal/humoral changes [4–8], alters renal sodium and water handling [9] and mediates transcriptional changes, occurring within hours, in the remaining non-ischemic kidney [10]. There is subsequent cell proliferation and hypertrophy followed by nephron hypertrophy and compensatory kidney growth [11]. Studies have reported that UNx increases blood flow in the remaining post-ischemic kidney, reduces intrarenal levels of vasoconstrictors, such as thromboxane A2, and lowers plasma renin activity. These changes might potentially reduce medullary circulatory dysfunction and hypoxia following IRI [12–14]. It has further been suggested that altered regional blood flow rather than maintenance of total renal blood flow (RBF), in the remaining kidney, is fundamental to the effect of UNx [12,13].

Research into the molecular mechanisms triggered by UNx in the remaining post-ischemic kidney has focused primarily on vasoactive substances [2,12–14]. This is in spite of several reports on the molecular response induced by UNx without superimposed IRI. Hence, UNx has been shown to down-regulate growth inhibitors and alters the balance between growth control suppressor and inducer elements, leading to proliferation and hypertrophy [10,11]. Proliferation of tubular epithelial cells is crucial for renal recovery following IRI [15]. UNx stimulates a wide range of growth factors such as IGF-1 [16,17], GH [18,19], HGF [20], VEGF [21] and EGF [22,23] with beneficial pleiotropic effects (e.g. proliferative, anti-apoptotic and tubulogenic as well as vasodilatory properties) [24–26].

The aim of this study was to investigate the effect of UNx on global and regional changes in renal filtration, perfusion and oxygenation as well as extracellular sodium regulation using non-invasive MRI technique both in the non-ischemic kidney and 24 hours after IRI, where persistent IRI-induced regional differences may be evident [27,28]. At this time point, repair and regenerative processes are active and may be further promoted by UNx. Using microarray technique, we aimed to identify differentially regulated genes following UNx, both in the non-ischemic and the ischemic setting. Our study revealed significant differences in regional perfusion as well as potentially renoprotective molecular mechanisms regulated by UNx. The myofibroblast may have a more prominent role in the very early inflammatory and fibrogenic response following renal IRI than earlier recognised.

## Material and methods

### Experimental animals

The national animal experiments inspectorate approved the study (number 2013−15−2934−00810). Standard housing conditions and husbandry practices were identical across the control and experimental groups. Adult male rats, *Rattus norvegicus*, *Wistar* (250–350 g) were supplied by Janiver Labs (Le Genest-Saint-Isle, France) and housed pairwise in cages, an artificial 12 h:12 h light-dark cycle and controlled temperature and humidity (21 ± 2°C and 55 ± 2%). The rats had free access to tap water and standard rodent chow (Altromin, Lage, Germany). The rats were cared for daily and monitored for pain and distress between and after the procedures in accordance to the general distress scoring sheet from Wolfensohn et al [29]. The rats were allowed to acclimate at least 1 week prior to the surgical procedures.

### Study design

Each animal underwent either right UNx or sham UNx, and was subjected to either 37 minutes of unilateral renal ischemia by left renal artery clamping or sham operation [30]. All operations were of similar total length. A total of 32 animals were randomized to the 4 groups as follows; sham (2 non-ischemic kidneys, n = 8), UNx (1 non-ischemic kidney, n = 8), IR (1 non-ischemic kidney, 1 post-ischemic kidney, n = 8) and IR+UNx (1 post-ischemic kidney, n = 8). One rat had to be replaced during surgery due to a renal cyst. Following 24 hours of reperfusion, the rat was anesthetized with sevoflurane and positioned in a MRI system, followed by harvesting of kidneys. The rats were terminated by cervical dislocation. Histopathologic assessment of renal tissue was performed in a blinded fashion. No further measures of blinding or randomization were taken. The study was designed and conducted according to international guidelines (S1 Text).

### Surgical procedures

Anesthesia was induced and maintained by inhalation of sevoflurane (2.5–5%) in O_2_. An injection of buprenorphine (Temgesic ®; RB Pharmaceuticals Limited, Berkshire, UK) 0.05 mg/kg was given s.c. in order to minimize postoperative pain. Buprenorphine administration was repeated every 8–12 hours until study end. Four mL of isotonic saline was administered s.c. at two different abdominal sites to avoid dehydration. The rat was placed on a heating pad, and a rectal probe was used to monitor and keep core temperature at 36.5–37.0°C. Prior to renal ischemia or sham ischemia, core temperature was allowed to stabilize at the target temperature.

The abdominal wall was opened using a median incision. First, the right-sided nephrectomy or sham procedure was performed. Next, the left renal artery was carefully dissected, and a non-traumatic microvascular clamp was used to occlude the artery to induce 37 minutes of warm ischemia [30]. The sham ischemia procedure involved visual identification of the left renal hilus. In all rats, a single droplet of lidocaine (10 mg/mL) was applied to the surgical area prior to renal artery dissection and 2 minutes prior to microvascular clamp removal. All post-ischemic kidneys resumed normal surface color within a preset limit of 3 minutes. Finally, the abdominal wall was closed in two layers using 4–0 absorbable sutures.

### MRI

MRI was performed 24 hours after induction of IRI using a 9.4 T preclinical MRI system (Agilent, Santa Clara, CA, USA) equipped with a dual tuned ^1^H/^23^NA volume rat coil (Doety Scientific, Columbia, SC). A tail vein catheter was inserted for contrast medium administration. During the MRI, the animal was kept under anesthesia with sevoflurane (3% sevoflurane, flow rate: 2 L/min). The body temperature was kept stable at 35–36°C, and respiration and capillary oxygen saturation were monitored. The MRI protocol included the following sequences: ^1^H T_2_‐weighted fast-spin-echo sequence, coronal and axial (repletion time (TR): 3 s, echo time (TE): 4 ms, flip angles: 90°/180°, echo train length (ETL): 4, echo spacing (ESP): 10 ms, FOV: 70 × 70 mm^2^, matrix: 128 × 128) for anatomical images. Blood-oxygen-level-dependent (BOLD) sequence (R_2_*-weighted) was performed using an axial ^1^H-multi-echo gradient-echo sequence, covering the entire abdomen with 32 slices. The sequence parameters were: TR: 800 ms, ΔTE: 2 ms, slice thickness: 2 mm, flip angle: 90°, matrix: 128 × 128, FOV: 80 × 80 mm^2^. A single-slice segmented Look–Locker sequence with a gradient-echo readout was used to acquire T_1_-weighted data, using the sequence parameters: TR: 3 ms, TE: 2 ms, inversion times (TI): 150, 250, 400, 600, 900, 1200, 2500, 4000 ms, flip angle: 8°, slice thickness: 2 mm, matrix: 128 × 128, FOV: 80 × 80 mm^2^. A dynamic contrast-enhanced (DCE) T_2_*-weighted sequence was performed using an axial ^1^H gradient-echo sequence, covering both kidneys in 1 slice, using the parameters: TR: 13.7 ms, TE: 1.85 ms, flip angle: 15°, slice thickness: 2 mm, matrix: 128 × 128, FOV: 60 × 60 mm^2^ with a single bolus of 0.05 mL of Gadoteric acid (Dotarem; 279.3 mg/mL; Guerbet, Villepinte, France) in isotonic saline (total injection volume of 1 mL and administered over 10 s).

Next, a sodium MRI was performed using ^23^Na gradient echo-sequence (TR: 60 ms, TE = 0.65 ms, flip angle: 90°, spectral width: 8 kHz, matrix: 32 x 32 x 8, FOV: 70 x 70 x 70 mm^3^.

**Fig 1:**
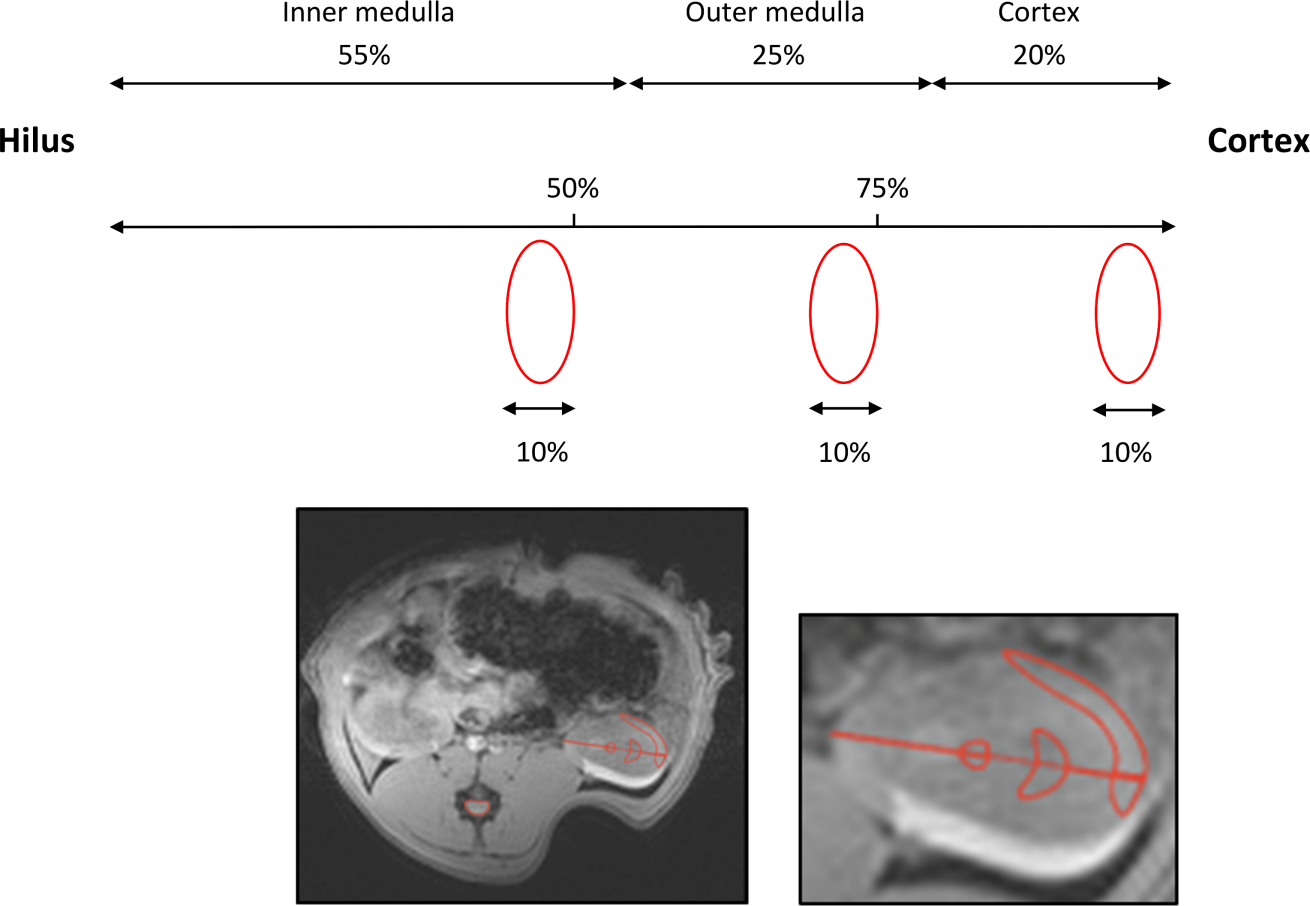
Regions of interest (ROIs). The size and position of the cortical, outer and inner medullary ROIs were approximated using the morphometrics of the different renal zones from the study by Oudar et al in male Wistar-Kyoto rats (mean body weight 220 g) [31]. The thicknesses of the zones in percent of the total kidney height along the corticopapillary axis were: cortex = 20%, outer medulla = 25% and inner medulla = 55%. First, the central part of the kidney was localized in the axial anatomical T2-weighted image. A vector was drawn starting from cortex ending at the very tip of the hilus/papilla, covering and measuring the whole kidney height. Two marks were placed in the direction cortex to hilus. The first was set to mark the outer medulla and covered 25% of the total kidney height. The second mark, the inner medulla ROI, was put in the centre and covered 50% of the kidney height. The height of each ROI was set to 10% of the total kidney height. The shape of the different zones on a HE-stained cross-section of the kidney were taken into consideration, when drawing the ROIs. The ROIs, we applied, are to some extent similar to the ones proposed by Oostendorp et al [28].

Data were imported and processed offline, where parametric maps of the relaxation times T_2_* and T_1_ were calculated. These maps and the ^23^Na image were converted to the file format Digital Imaging and Communications in Medicine (DICOM). Regions of interests (ROIs) were drawn and analyzed using OsiriX (Pixmeo SARL, Bernex, Switzerland) (Fig 1). DCE images were processed and analyzed using Mistar software (Apollo Medical Imaging Technology, Melbourne, Australia), in which measures of RBF and glomerular filtration rate (GFR) were calculated based on a two-compartmental kinetic model.

### Renal functional measurements

Plasma creatinine (P_cr_) and urea (P_urea_) were measured using the Roche Cobas 6000 analyser (Roche Diagnostics, Basel, Switzerland).

### Tissue handling

Kidneys were kept on ice and cut into two equal pieces along the short axis. One half was fixed in phosphate buffered 4% paraformaldehyde for 2–4 hours, washed and stored in phosphate buffered saline-Tween (PBS-Tween) at 4°C until further tissue preparation. The other half was divided into three parts: 1) cortex, 2) cortex and outer medulla, and 3) inner medulla, and snap-frozen in liquid nitrogen. Blood samples were collected in EDTA tubes and centrifuged for 10 minutes at 3000 × g. Kidney and plasma samples were stored at -80°C until further processing.

### Histological examination

Fixed kidney tissue (all zones represented, including the renal papilla) was dehydrated, paraffin-embedded and cut in slices of 2-μm on a rotary microtome (Leica, Microsystems A/S, Herlev, Denmark). Then tissue was stained with the periodic acid-Schiff (PAS) stain. The histopathologic damage in cortex and outer medulla was evaluated by a board certified pathologist, SK using conventional light microscopy.

### RNA isolation

The kidney tissue was homogenised in lysis buffer (Macherey Nagel, Düren, Germany) on a TissueLyser LT (Qiagen, Venlo, Netherlands) for 30 seconds at 1250 rpm and centrifuged at 1000 × g for 10 minutes at 4°C. Total RNA was obtained using an RNA Isolation Kit (Macherey Nagel) in accordance with the manufacturer’s instructions. The RNA concentration and purity were determined spectrophotometrically. The same batch of total RNA, containing both renal cortex and outer medulla, was used both for microarray analysis and confirmatory QPCR.

### Microarray analysis

Samples from three animals from each group were randomly selected for microarray analysis performed by a commercial transcriptomics provider (Aros Applied Biotechnology, Eurofins, Aarhus, Denmark) using the Affymetrix Clariom D array cartridge (Thermo Fischer, Santa Clara, CA, USA) according to standard procedures. cDNA was synthesized from 150 ng RNA, transcribed in vitro, and labelled utilizing the WT PLUS reagent kit. The generated target RNA was loaded onto the array cartridge and hybridized for 16 hours at 45°C. After washing and staining in the GeneChip Fluidics Station, the array was scanned in the GeneChip Scanner 7G. The image files were imported into the Affymetrix Expression Console software. Background correction and quantile normalization were performed after quality control of the data. The experimental data have been deposited in the NBCI Gene Expression Omnibus (GEO) under series entry GSE100540.

### Gene expression analysis

The Affymetrix Clariom D array detects all known, coding rat genes. Normalized gene expression data was analysed in the open source software Multiexperiment Viewer version 4.9.0, which is part of the TM4 microarray software suite [32]. The statistical analysis was done by employing the Significance Analysis of Microarrays (SAM)-method [33]. The number of permutations was set to 200, S0 (the exchangeability factor, which was set to the *Tusher et al method* [33]), and the k-nearest neighbours method for imputation of missing data with k = 10 was used. Unpaired two-class analysis was performed. A false discovery rate (FDR) of 10% was selected in the functional enrichment and specific pathway analysis. Functional enrichment analysis was done by using the DAVID Bioinformatics Resources 6.8 [34]. The following criteria defined the threshold of an enriched category: a modified Fischer Exact p-value of < 0.05 (> 1.3 after -log10 transformation), and n ≥ 2 genes represented in a category. For further pathway analysis the Ingenuity ® Pathway Analysis (IPA) software (Qiagen Bioinformatics, Hilden, Germany) was used.

### Quantitative polymerase chain reaction

cDNA was synthesised using a RevertAid First Strand cDNA synthesis kit (Thermo Fisher Scientific, Waltham, MA, USA). Samples were made using Maxima SYBR Green QPCR Master Mix (Thermo Fisher Scientific). A standard curve was generated by mixing an equal amount of cDNA from each experimental group, diluting the mixture successively and applying it into separate wells. The QPCR protocol consisted of 40 cycles of denaturation (30 seconds at 90°C), annealing and synthesis (60 seconds at 60°C). The primers are shown in supplementary files (S1 Table). *Gapdh* was used for normalisation of the target gene.

### Statistical analysis

Data are expressed as mean ± standard error of mean (SEM). Normality was checked by a quantile plot and histogram. Equality of standard deviations was checked by Bartlett’s test. Data were ln-transformed, when necessary. Comparison of means between groups was performed with two-way analysis of variance (ANOVA) with Bonferroni's multiple comparisons post-test or the Kruskal-Wallis test with Dunn’s multiple comparisons post-test. Comparison of means within a group (MRI-data) was performed by paired one-way ANOVA or Friedman's test with Bonferroni’s or Dunn’s multiple comparisons post-tests. The comparison of the means of right and left kidney weights in the IR group was performed using the Wilcoxon matched-pairs signed rank test since these data did not meet the assumption of normal distribution. Post-hoc analysis of differences in histopathologic injury between the two ischemic groups was performed using Student’s t-test. P values < 0.05 were considered statistically significant. Data were analysed using STATA version IC/13.1 for Windows (StataCorp LP, College Station, TX, USA) and GraphPad Prism version 6.05 for Windows (GraphPad Software, San Diego, CA, USA). Sample size calculation was based on a pilot study, where UNx attenuated the IRI-induced increase in *Tnf* mRNA.

## Results

All animals survived until planned termination at the study end. IRI significantly increased kidney weight in both the IR and the IR+UNx group compared with the sham group (Table 1). As expected the IR+UNx group demonstrated a marked rise in P_cr_ and P_urea_ compared with the IR group. UNx significantly reduced renal histopathologic injury, e.g. epithelial necrosis and outer medullary casts, after IRI (Table 2).

Two animals from the IR+UNx group were excluded from all analyses due to a hemorrhagic infarction in the kidney.

**Table 1:** Physical parameters, plasma and arterial gas analysis parameters.

	*Sham*	*UNx*	*IR*	*IR+UNx*
BW [g] (baseline)	295 ± 11.5	305 ± 10.5	296 ± 8.4	305 ± 12.2
Δ BW [g]	-4.0 ± 6.1	1.9 ± 4.3	3.8 ± 2.8	5.7 ± 2.9
KW [g/100 g BW]	0.33 ± 0.01	0.38 ± 0.02	0.43 ± 0.014 (LK) *** 0.38 ± 0.012 (RK) $	0.40 ± 0.01 *
P_cr_ [μmol/L]	26 ± 1.9	41 ± 1.6 *	39 ± 1.7	198 ± 42.6 ****, #
P_urea_ [mmol/L]	6 ± 0.2	8 ± 0.3	8 ± 0.3	25 ± 4.1 ****
P_O2_ [kPa]	8.4 ± 0.6	9.2 ± 0.9	9.5 ± 0.6	11.0 ± 0.9
Hb [mmol/L]	7.3 ± 0.3	7.7 ± 0.1	8,2 ± 0.3	7.6 ± 0.1
Hct [%]	36 ± 1.4	38 ± 0.6	40 ± 1.6	37 ± 0.6
S_O2_ [%]	78 ± 4	80 ± 5	83 ± 3	88 ± 1

Values are presented as means ± SEM. Measurements are from the day of termination unless otherwise stated. Comparison of unpaired group means was performed using either two-way ANOVA followed by Bonferroni’s multiple comparisons post-test or Kruskal-Wallis test with Dunn's multiple comparisons post-test.

*, P < 0.05

***, P < 0.005

****, P < 0.0001 vs. sham

#, P < 0.05 vs. IR. Comparison of paired group means was performed by Wilcoxon signed rank test.

$, P < 0.01 vs. left kidney (LK).

BW, body weight; KW, kidney weight; P_cr,_ plasma creatinine; P_urea,_ plasma urea; P_O2,_ partial pressure of oxygen; Hb, hemoglobin; Hct: hematocrit; S_O2,_ oxygen saturation; UNx, unilateral nephrectomy; IR, ischemia-reperfusion. Number of animals: sham, UNx and IR groups (n = 8), IR+UNx (n = 6).

**Table 2:** Effect of unilateral nephrectomy on renal histologic damage after ischemia-reperfusion injury.

	*Sham*	*UNx*	*IR*	*IR+UNx*
Cortical casts	0	0	1.2 ± 0.17	1.6 ± 0.18
Epithelial necrosis	0	0	3.6 ± 0.18	2.7 ± 0.42 *
Tubular dilatation	0	0	1.5 ± 0.19	1.0 ± 0.26
Epithelial cell detachment	0	0	3.3 ± 0.25	2.3 ± 0.42
Edema	0	0	0	0
Inflammation	0	0	0.13 ± 0.13	0.17 ± 0.17
Outer medullary casts	0	0	4.0 ± 0	2.8 ± 0.48 *

Values are presented as means ± SEM. Histopathologic scores for each item ranged from 0–4 depending on the size of the affected area: 0: < 1%, 1: 1–5%, 2: 5–25%, 3: 25–50% and 4: 50–100%. Evaluation was done by co-author SK, who is a board certified pathologist. Comparison of IR and IR+UNx group means was performed as a post-hoc analysis using Student's t-test.

*, P < 0.05 compared with IR.

UNx, unilateral nephrectomy; IR, ischemia-reperfusion. Number of animals: sham, UNx and IR groups (n = 8), IR+UNx (n = 6).

### Unilateral nephrectomy enhances perfusion in the inner medulla

**Fig 2:**
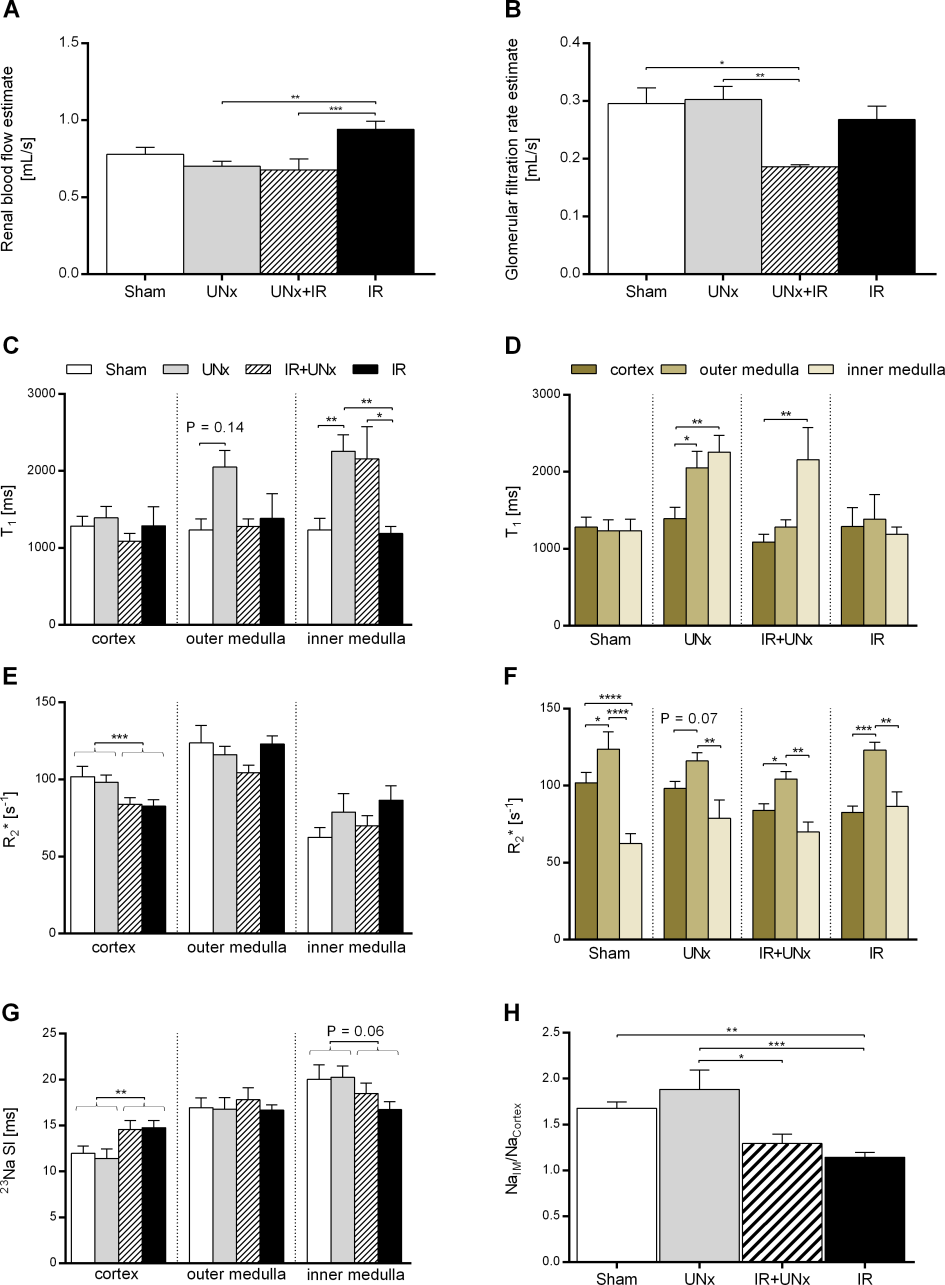
MRI experiments. A: Estimates of renal blood flow and B: glomerular filtration rate at 24 hours both adjusted for kidney size. The parameters are based on dynamic contrast enhanced magnetic resonance imaging (DCE-MRI) and analysis using the Patlak-Rutland plot. Intrarenal T_1_, R_2_* and ^23^Na values presented D+F: groupwise and C+E+G: from the different regions of the kidney: the cortex, outer medulla and inner medulla. H: The ratio between sodium signal intensity in inner medulla normalized to cortex, representing the cortico-medullary sodium signal gradient. All values are expressed as mean ± SEM. The effect of UNx and IRI were evaluated by two-way analysis of variance (ANOVA). Group means were compared using two-way ANOVA followed by Bonferroni’s multiple comparisons post-test or the Kruskal-Wallis test with Dunn’s post-test. Means within a group were compared using paired one-way ANOVA or Friedman’s test.* P < 0.05, ** P < 0.01, *** P < 0.005 and **** P < 0.0001. Number of animals: Sham (DCE, n = 7; T_1_, n = 6; BOLD + ^23^Na, n = 8), UNx (DCE, n = 7; T_1_ + BOLD + ^23^Na, n = 8), IR (n = 8) and IR+UNx (DCE-MRI, n = 5; T_1_ + BOLD + ^23^Na, n = 6). IR, ischemia-reperfusion; UNx, unilateral nephrectomy.

DCE-MRI was applied to assess kidney perfusion and filtration. The DCE-MRI estimates of RBF and GFR are shown in Fig 2A and 2B. IRI reduced GFR in the IR+UNx and IR groups compared with the sham group (IR+UNx, P < 0.05; IR, P = NS). Neither IRI nor UNx decreased RBF compared with sham. However, RBF was increased in the IR group compared with the IR+UNx group (P < 0.001). To examine regional perfusion and oxygenation in the kidney, slice-selective T_1_- and BOLD-weighted sequences with parametric mapping were used. Fig 2C shows calculated T_1_ values in the different zones of the kidney. The T_1_ was 66% (P = 0.14) and 83% (P < 0.01) greater in the outer and inner medulla, respectively, in the UNx group compared with the sham group. The T_1_ value was significantly higher in outer medulla compared with cortex within the UNx group (Fig 2D). The inner medulla T_1_ was significantly higher (82%) in the IR+UNx group compared with the IR group. The T_2_*(BOLD)-weighted MRI is sensitive to changes in the amount of deoxygenated Hb per voxel, and R_2_* (1/T_2_*) is generally regarded to relate linearly to the Hb concentration. In the sham group, the R_2_* value was significantly lower in inner medulla compared with cortex (Fig 2F). The cortical R_2_* value was significantly decreased by IRI, indicating a higher oxygenation in cortex after IRI. We used a ^23^Na-MRI sequence to investigate possible changes in the renal cortico-medullary sodium gradient following UNx. As shown in Fig 2G, the ^23^Na signal intensity increased gradually from cortex to inner medulla in all groups. In Fig 2H, the inner medullary ^23^Na signal intensity normalized to cortex is presented. IRI significantly reduced this gradient. We have summarized main results in Table 3.

**Table 3:** Changes in outcome measures.

	*UNx vs sham*	*IR vs sham*	*IR+UNx vs sham*	*IR+UNx vs IR*
DCE-MRI, GFR [mL/s]	↔	↔	↓	↔
DCE-MRI, RBF [mL/s]	↔	↔	↔	↓
Slice-selective T_1_-weighted-MRI, T_1_ [ms]	↑	↔	↔	↑
BOLD-MRI, R_2_* [s^-1^]	↔	↓	↓	↔
^23^Na-MRI, CM sodium gradient	↔	↓	↓	↔
Morphologic injury	↔	↑	↑	↓
Pro-inflammatory gene expression	↔	↑	↑	↓
Pro-fibrotic gene expression	↔	↑	↑	↓

Arrow indicates if the change was positive or negative compared with sham or control ischemic group, or if no change was present. CM, cortico-medullary. Please refer to Figs 2 and 5–7 for details.

### Unilateral nephrectomy mainly up-regulates genes in the remaining non-ischemic kidney, whereas it mainly down-regulates genes in the remaining post-ischemic kidney

**Fig 3:**
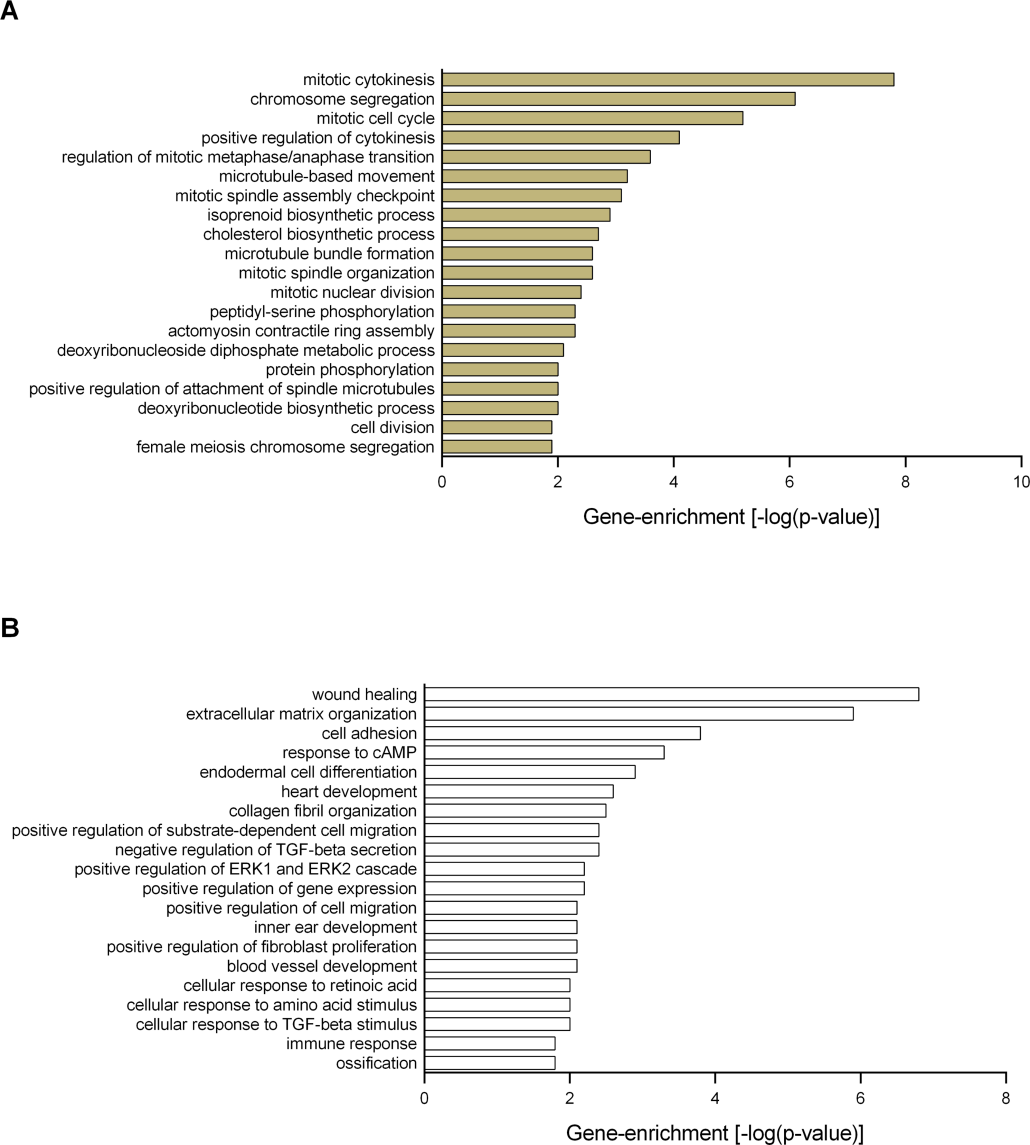
Functional enrichment analysis. The Significance Analysis of Microarrays (SAM) identified A: 56 up-regulated genes (without IRI) and B: 47 down-regulated genes (with IRI) in response to UNx. Functional enrichment analysis was performed using these gene sets in DAVID Bioinformatics Resources, and the enriched Gene Ontology annotations (the biological processes) are presented. The following criteria defined the threshold of an enriched category: a modified Fischer Exact p-value of < 0.05 (> 1.3 after -log10 transformation), and n ≥ 2 genes represented in a category. Number of animals, n = 3 in each group.

S1 Fig displays the total number of differentially regulated genes identified by unpaired two-class SAM analysis. The 56 genes differentially regulated by UNx were exclusively up-regulated compared with the sham treatment. Gene enrichment analysis showed that the most highly enriched biofunctional categories were mitotic cytokinesis, chromosome segregation and mitotic cell cycle (Fig 3A).

UNx significantly down-regulated the expression of 47 genes in the setting of IRI. Gene enrichment analysis showed that UNx significantly down-regulated genes involved in wound healing, ECM organization and cell adhesion as well as positive regulation of fibroblast proliferation and immune response (Fig 3B and S2 Table). UNx was associated with suppression of genes encoding different types of collagen (*Col1a2* and *Col12a1*), collagen triple helix repeat containing 1 (*Cthrc1*), tenascin C (*Tnc*), fibulin- 1 (*Fbln1*) and fibronectin-1 (*Fn1*). In addition, *Acta2* (also known as *α-Sma*, actin, alpha 2, smooth muscle, aorta), which is expressed by differentiated myofibroblasts, was down-regulated. The above mentioned genes were all significantly induced by IRI. The top differentially regulated genes are presented in supplementary files (S3 Table).

### UNx down-regulates the expression of pro-fibrotic genes in the remaining post-ischemic kidney

Pathway analysis was performed to investigate the possible underlying molecular mechanisms responsible for the observed suppression of fibrogenic genes.

**Fig 4:**
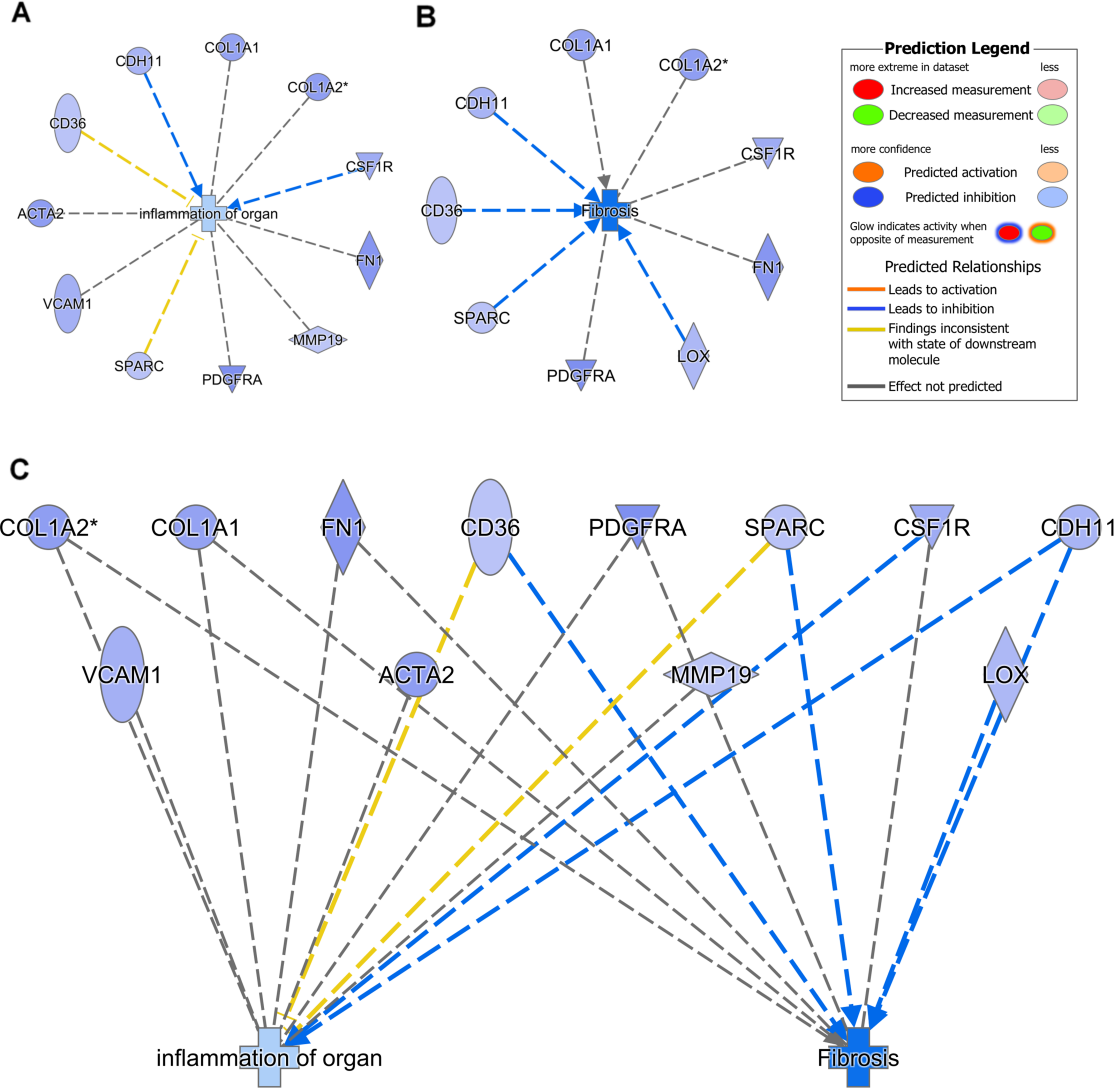
Unilateral nephrectomy suppresses genes associated with fibrosis and inflammation. Pathway analysis was done using Ingenuity ® Pathway Analysis (*the diseases and biofunctions* application). Unilateral nephrectomy was predicted to inhibit the A: fibrosis and B: inflammation processes following renal ischemia-reperfusion injury by down-regulating the displayed genes. C: Genes involved in both fibrosis and inflammation are identified. Number of animals, n = 3 in each group.

S2A Fig shows the most highly enriched canonical pathways. UNx down-regulated the genes in the pathway hepatic fibrosis/hepatic stellate cell activation such as platelet derived growth factor receptor alpha (*Pdgfra*). UNx also suppressed genes associated with the inflammatory response (complement system, agranulocyte adhesion and diapedesis and granulocyte adhesion and diapedesis, dendritic cell maturation). Pathway analysis identified the most significantly regulated network of molecules (S2B Fig). Next, we aimed at exploring differentially regulated genes associated with fibrosis and inflammation and the possible interactions with *the diseases and biofunctions* application in IPA. UNx significantly suppressed genes in the biofunctions: fibrosis and inflammation of organ (Fig 4, 4A, 4B and 4C).

### QPCR confirms that UNx significantly suppresses fibrosis and inflammation-associated genes

**Fig 5:**
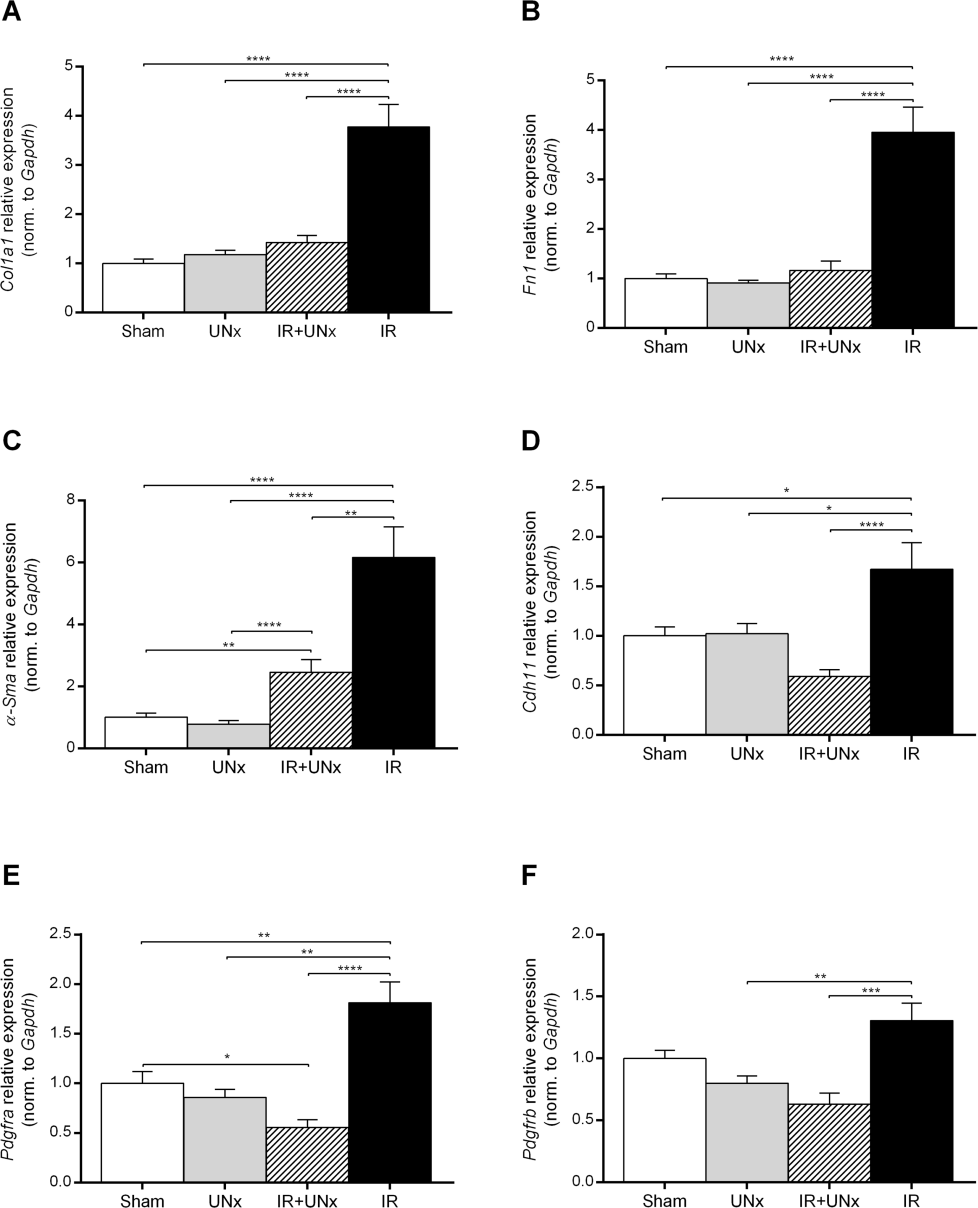
Validation of fibrosis-associated genes. A: *Col1a1*, collagen type 1 alpha 1 chain. B: *Fn1*, fibronectin-1. C: *α-Sma*, actin, alpha 2, smooth muscle, aorta. D: *Cdh11*, cadherin-11. E and F: *Pdgfra* and *Pdgfrb*, platelet derived growth factor receptor alpha and beta. Bars represent mRNA levels by quantitative polymerase chain reaction in the left renal cortex and outer medulla normalised to *Gapdh* mRNA. All values are expressed as mean ± SEM. Group means were compared using two-way ANOVA followed by Bonferroni’s multiple comparison post-test. * P < 0.05, ** P < 0.01, *** P < 0.005 and **** P < 0.0001. Number of animals: sham, UNx and IR groups (n = 8), IR+UNx (n = 6). IR, ischemia-reperfusion; UNx, unilateral nephrectomy.

We validated microarray results by QPCR in relation to the expression of genes associated with fibrosis, macrophages and immune mediators. IRI was associated with a significant increase in the expression of *Col1a1* and *Fn1* compared with the sham treated animals (Fig 5, 5A and 5B), while UNx abolished this increase. IRI was associated with a significant increase in the expression of *α-Sma* compared with the sham group, and UNx decreased this expression significantly compared with the IR group (Fig 5C). Cadherin-11 (*Cdh11*) was significantly up-regulated in the IR group, but not in the IR+UNx group (Fig 5D). The expression of *Pdgfra* was also significantly increased in the IR group (Fig 5E), and this was attenuated by prior UNx (P < 0.0001). The *Pdgfrb* gene demonstrated a similar expression pattern (Fig 5F)

**Fig 6:**
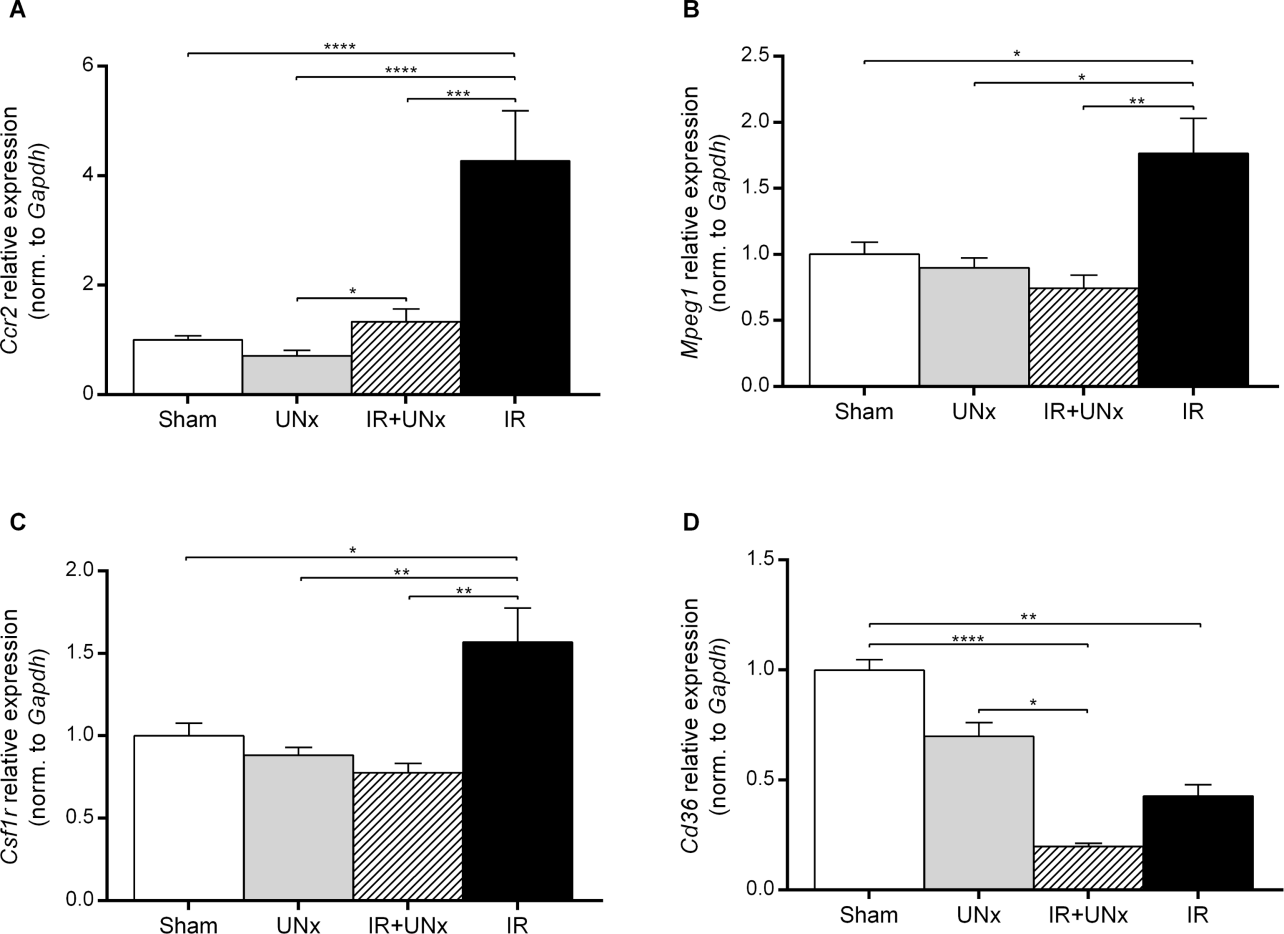
Validation of macrophage expressed genes. A: *Ccr2*, C-C chemokine receptor type 2. B: *Mpeg1*, macrophage expressed 1. C: *Csf1r*, colony stimulating factor 1 receptor. D: *Cd36*, cd36 molecule. Bars represent mRNA levels by quantitative polymerase chain reaction in the left renal cortex and outer medulla normalised to *Gapdh* mRNA. All values are expressed as mean ± SEM. Group means were compared using two-way ANOVA followed by Bonferroni’s multiple comparison post-test. * P < 0.05, ** P < 0.01, *** P < 0.005 and **** P < 0.0001. Number of animals: sham, UNx and IR groups (n = 8), IR+UNx (n = 6). IR, ischemia-reperfusion; UNx, unilateral nephrectomy.

**Fig 7:**
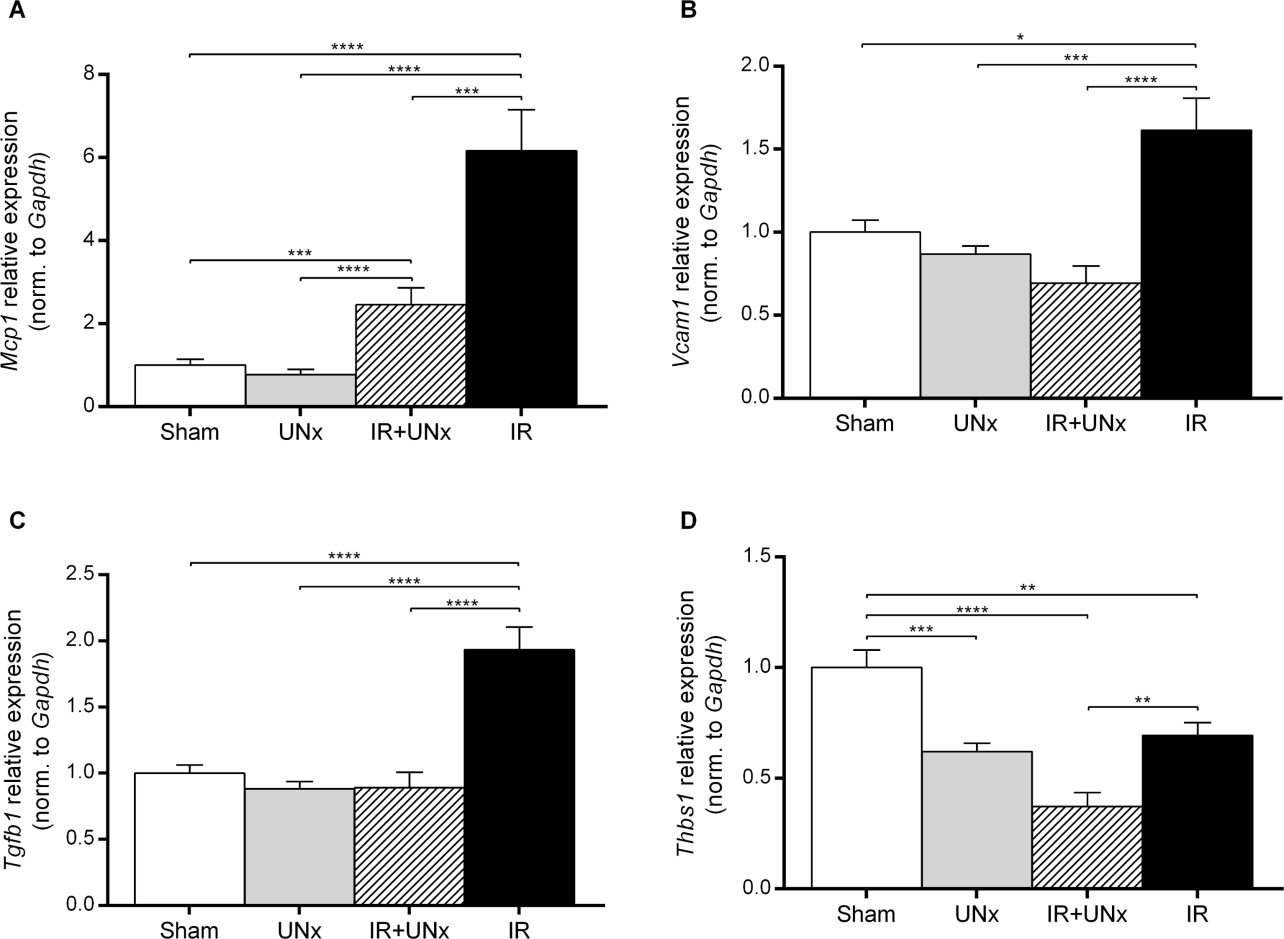
Validation of inflammatory mediators genes. A: *Mcp1*, monocyte chemoattractant protein-1. B: *Vcam1*, vascular cell adhesion molecule-1. C: *Tgfb1*, transforming growth factor-β1. D: *Thbs1*, thrombospondin-1. Bars represent mRNA levels by quantitative polymerase chain reaction in the left renal cortex and outer medulla normalised to *Gapdh* mRNA. All values are expressed as mean ± SEM. Group means were compared using two-way ANOVA followed by Bonferroni’s multiple comparison post-test. * P < 0.05, ** P < 0.01, *** P < 0.005 and **** P < 0.0001. Number of animals: sham, UNx and IR groups (n = 8), IR+UNx (n = 6). IR, ischemia-reperfusion; UNx, unilateral nephrectomy.

The transcript level of the C-C chemokine receptor type 2 (*Ccr2*) gene expressed by monocyte/macrophages [35] was attenuated by prior UNx (P < 0.005) (Fig 6A). Transcript levels of both *Mpeg1* (macrophage expressed-1, also known as Perforin-2) and *Csf1r* (colony stimulating factor 1 receptor) [36] were significantly down-regulated in the IR+UNx group compared with the IR group (Fig 6, 6B and 6C). Expression level of monocyte chemoattractant protein-1 (*Mcp1*) was significantly increased in the IR group compared with sham, and attenuated by UNx (Fig 7A). UNx blocked the up-regulation of the vascular cell adhesion molecule-1 (*Vcam1*) (P < 0.0001, Fig 7B) observed in relation to IRI. Interestingly, *Tgfb1* was significantly increased by IRI in the non-UNx group, and this increase was significantly attenuated by UNx (Fig 7C). UNx down-regulated thrombospondin-1 (*Thbs1*) compared with the sham group both in the non-ischemic kidney (P < 0.005) and the post-ischemic, contralateral kidney (P < 0.0001, Fig 7D). *Thbs1* was also suppressed by IRI in the non-UNx group (P < 0.01). The mRNA level of *Cd36* was suppressed in the UNx group and the IR group compared with sham (P = 0.47, P < 0.01), but was further down-regulated by the combination of UNx and IR (P < 0.0001, Fig 6D).

## Discussion

The main findings of our study are that 1) UNx reduced ischemic histopathologic injury both in cortex and outer medulla of the remaining, contralateral kidney, 2) this could be attributed to UNx-mediated increase in medullary perfusion and 3) a decreased pro-inflammatory response. It may be speculated that UNx reduces post-ischemic tissue injury and expression of pro-inflammatory and pro-fibrotic genes by improving medullary perfusion. On the other hand, it is also possible that UNx facilitates vasodilatation and improved perfusion by reducing the production of pro-inflammatory cytokines such as *Tnf*.

### Alterations in hemodynamics, oxygenation and sodium handling following UNx with and without IRI

This study demonstrated that UNx increases perfusion in the inner medulla in both the remaining non-ischemic and post-ischemic kidney. To our knowledge, this is the first report on the changes in regional perfusion following UNx with superimposed IRI. A number of experimental IRI studies have found that blood flow to the outer medulla is persistently and disproportionately (relative to RBF reduction) reduced in the reperfusion phase, aggravating tissue injury in this area [37,38]. Previous experimental studies have found that UNx reduces renal dysfunction and tissue injury 24–48 hours to 2 weeks after IRI [2,3,12–14,39–41] In general, the improved GFR is associated with an increased RBF (recovery or absolute) [12–14,39–41], but it is not always the case [3,12,13]. Furthermore, UNx does not always promote increased GFR in the post-ischemic kidney, even when RBF is maintained (at 160 min into reperfusion) [41]. Improved renal function may be caused by diminished structural injury instead, which we did detect. Interestingly, it has been speculated that altered regional blood flow rather than maintenance of total RBF is fundamental to the effect of UNx [12,13]. Our main finding is that UNx increases medullary perfusion. It is possible that intrarenal redistribution of blood flow is a prerequisite for the observed increase in inner medullary perfusion. Such mechanism is supported by Moskowitz et al. [42], demonstrating that blood flow is redistributed from renal cortex to renal medulla within 1 hour after UNx. This seems possible since we also noted that UNx increased perfusion in both inner and outer medulla in the non-ischemic kidney compared with cortex. However, we did not detect a concomitant drop in cortical perfusion in the UNx groups, implying that the increase in medullary perfusion theoretically would need to be dependent on an increase in total RBF. Nevertheless, our data showed that the RBF was unaffected 24 hours after UNx compared with the sham group, albeit lower than the IR group. Previous studies have shown that RBF increased in the remaining non-ischemic kidney following UNx [12,13,43,44] (30 minutes to 6 weeks post-nephrectomy) or the remaining ischemic kidney [12–14,39–41] (2 hours to 2 weeks after IRI). This discrepancy between studies may relate to differences in assessment method, since neither of the reported studies used MRI, model-specific factors (ischemia time, species/strain differences, etc.) and/or differences in the time point of measurements. In our study, UNx did not cause an increase in the MRI-based GFR estimate in the remaining non-ischemic kidney at 24 hours of reperfusion. This finding is in contrast to the majority of previous experimental studies and clinical reports from living kidney donors [12,13,45]. IRI only lead to a statistical significant reduction in GFR in the IR+UNx group in which large increases in P_cr_ and P_urea_ were also observed. However, we previously showed that even 60 minutes of unilateral ischemia, normally giving rise to severe AKI, only gives rise to minor increases in P_cr_ and P_urea_ 24 hours after ischemia, when a normal contrateral kidney is left in situ [46]. In addition, both DCE-MRI and the slice-selective T_1_-sequence are known to have limitations, which could explain the inconsistencies in our data [47]. The slice-selective T_1_-sequence has the advantage of having a high spatial resolution that enables the detection of signal intensity differences in the renal compartments, but the calculated value is obviously influenced by many factors, including perfusion, tissue composition and water content.

Sodium reabsorption in the remaining kidney will decrease acutely following UNx in order to maintain sodium homeostasis, potentially lowering oxygen demand. We did not find any significant effect of UNx on sodium signal intensity in any of the renal zones. We did, however, as expected, observe IRI-induced attenuation of the cortico-medullary sodium gradient. Similar results have been reported in the early phase of reperfusion (10–60 minutes after index ischemia) and 6 hours after induction of AKI by nephrotoxic chemicals [48,49]. Tubular transport and the countercurrent exchange system are compromised during IRI, decreasing osmolarity in the medulla. Our data extends the findings in showing that the sodium signal intensity gradient is also disrupted 24 hours after reperfusion. In addition, our data show that the diminished sodium gradient seems to be a result of lower sodium signal intensity in inner medulla as well as a higher level in cortex. Atthe et al. found that relaxation rates in cortex and medulla were equally affected by IRI. This supports the use of sodium signal intensity as a proxy for sodium concentration in evaluating cortico-medullary gradient differences between non-ischemic and ischemic kidneys [48]. It can be speculated that the increased sodium signal intensity in cortex and the decreased level in the inner medulla could be a compensatory shift in reabsorption of sodium to cortical proximal convoluted tubules, compensating for impaired tubular reabsorption in the more damaged medullary straight portion of the proximal tubules and thick ascending limb of the loop of Henle that compromises the countercurrent mechanism in the first place [49]. However, we cannot rule out that it is caused by changed relaxation rates in out setup, morphologic injury and edema [50], since tubular sodium reabsorption is in general compromised by IRI through a reduction in tubular sodium transporters [51]. We observed a concomitant decrease in the R_2_* in the cortex of the post-ischemic kidney compared with non-ischemic kidneys. This could be explained by the volume effect of edema on the R_2_* parameter. In line with our results, Oostendorp et al. also found a lower R_2_* value in cortex of ischemic kidneys 24 hours after reperfusion suggesting a higher oxygenation [28]. In inner medulla, the R_2_* value was not significantly different comparing the UNx groups to non-UNx groups, even though we observed an increased T_1_ value. It may be possible that oxygen consumption is correspondingly increased in the UNx groups, thus tending to increase the R_2_*. Outer medullary hypoxia has been demonstrated after IRI, by BOLD-MRI, in both the immediate reperfusion phase (up to 100 min) and 24 hours after reperfusion [28,52]. Our results could not confirm this. However, we did detect a low R_2_* in inner medulla in the sham group. This finding is similar to other studies and probably caused by increased water content, lower vascular volume and Hct per tissue volume [53].

In summary, we showed that UNx enhanced perfusion in the inner medulla of the kidney. This finding may play a role in the overall protection afforded by UNx against IR-induced kidney injury. However, UNx did not significantly change the renal oxygenation or the cortico-medullary sodium gradient as assessed by BOLD and ^23^Na-MRI. In contrast to previous investigations, UNx did not increase GFR or RBF.

### The transcriptional response to UNx with and without IRI

Under normophysiologic states, UNx induced the expression of genes associated with cell proliferation, whereas UNx suppressed several genes encoding structural ECM components in the post-ischemic kidney.

### The transcriptional response to UNx without superimposed IRI

The gene enrichment analysis showed that UNx induced genes that serve important functions during cell cycle progression and mitosis. The most highly enriched biofunctional categories were mitotic cytokinesis, chromosome segregation and mitotic cell cycle. These functions are part of the processes exclusively taking place in the M phase of cell cycle. For instance, UNx induced *Plk-1* (polo-like kinase 1), *Cdk-1* (cyclin-dependent kinase 1) and *Ki-67* (marker of proliferation Ki-67). This highly indicates that UNx induces cell proliferation in the remaining kidney at 24 hours after UNx. Compensatory kidney growth was observed in 79.3% of the cases 6–12 months after human kidney donation [54]. Our study supports the results of other studies showing that the gene transcription pattern is altered acutely (at 1 hour) following UNx [10]. In contrast to our data, compensatory kidney growth was promoted by down-regulation of growth inhibition elements at 24 hours in the study by Hauser et al. [11]. We found no changes in the expression of various growth factors previously implicated in compensatory kidney growth after UNx [17–21,23]. The proliferative response induced by UNx could be beneficial for the repair and regeneration of injured tubules.

### The transcriptional response to UNx with superimposed IRI

The most abundantly suppressed genes by UNx belonged to the biological group of structural ECM components such as different types of collagen (*1a2*, *8a1*, *1a1*, *12a1* and *6a3*), *Fn1*, *Fbln1* and *fibrillin-1*. An interesting finding is that UNx also reduced the expression of *α-Sma* and *Cdh11* expressed by (activated) myofibroblasts [55]. We validated the microarray data and explored the underlying molecular mechanisms. We demonstrate that UNx diminished the IRI-induced increase in expression of representative genes encoding structural ECM components *Col1a1* and *Fn1* at 24 hours of reperfusion. Chronically activated and highly *α-Sma* expressing myofibroblasts are the dominant cell type expressing *Col1a1* and *Fn1* in the late fibrotic stage of ischemic kidney injury. Pericyte to myofibroblast differentiation has been implicated as an important source of myofibroblasts in the injured kidney [56]. Their role in the renal repair process, starting within 24 hours after the ischemic insult, is less well described. The elevated *Cdh11* supports that myofibroblasts are the primary cells expressing *α-Sma* in our study. *Cdh11* is a cell-cell adherens junction, highly expressed by activated (*α-Sma*-positive) myofibroblasts in the skin [55]. Interestingly, *Cdh11* was recently found to be increased in both renal tissue and urine in CKD animal models (including IRI) and in patients with CKD [57,58]. We document that IRI indeed induces *α-Sma* and *Cdh11* simultaneously, suggesting that myofibroblast proliferation and activation is triggered by IRI already at 24 hours of reperfusion. This finding suggests that myofibroblasts may also play a role in the early injury phase after renal IRI. IRI induced the expression of monocyte/macrophage genes such as *Ccr2*, *Mpeg1* and *Csfr1r*, which indicates the presence of activated macrophages recruited into the injured kidney. Our results showed that UNx is associated with reduced expression of these genes, indicating reduced infiltration of macrophages. In addition, UNx reduced the expression of *Pdgfra*. Most importantly this is in line with a previous report by Hauser et al. [11]. Nakagawa et al. noted that *Pdgfra* and -*b* transcript levels were up-regulated acutely after 50 minutes of bilateral renal artery clamping [59]. We observed induction of the *Pdgfra* and -*b* in the post-ischemic kidney, but only in the IR group. Interestingly, Chen et al. showed that both Pdgfr-subunit transcripts were up-regulated in the kidney from 1–14 days after unilateral ureteral obstruction (UUO), the α-subunit exclusively being expressed by pericytes [60]. Both specific and dual blockade of the Pdgfrs inhibited pericyte proliferation and differentiation into myofibroblasts, *Mcp1* transcript level, macrophage infiltration and *Tgfb1* expression at 4 days and fibrosis at 14 days following UUO. This highlights the pericyte/myofibroblast as a central mediator of inflammation and fibrogenesis. Our data, though only assessed on the transcriptional level, suggested that UNx may diminish the IRI-induced proliferation of pericytes and myofibroblasts, in turn reducing the expression of ECM components. This may have additional immunomodulatory effects due to lowering of the pericyte/myofibroblast contribution to *Mcp1* (protein) production.

Reduced myofibroblast and ECM-gene expression could also be related to the observed attenuated *Tgfb1* levels. *Tgfb1* (protein) induces proliferation and activation of fibroblasts and myofibroblasts [61]. *Tgfb1* (protein) is produced by a variety of cells during IRI, including pro-fibrotic subtypes of macrophages, e.g. the *Cd36* positive macrophages [62–65]. In our model, the decrease in myofibroblast markers and particularly in attenuated ECM gene expression could partially be explained by diminished macrophage infiltration in the kidney, including *Cd36* positives. LPS depletion of macrophages attenuated persistent inflammation and fibrosis 4–8 weeks after IRI [66] UNx was associated with a significant reduction in *Thbs1* expression. Inhibition of *Thbs1* (protein) has been shown to be renoprotective against IRI-induced tubular damage and inflammation [67,68], potentially mediated by less activation of the latent *Tgfb1* (protein) [69]. Interestingly, our microarray results also revealed that UNx attenuated the rise in connective tissue growth factor (*Cnn2*), a matricellular protein (non-structural ECM protein) with multiple pro-fibrotic functions. *Cnn2* (protein) is secreted by fibroblasts and is both a potent activator of myofibroblasts, but also a co-factor for *Tgfb1* (protein) binding to its receptor [55,70]. UNx was associated with the down-regulation of additional matricellular protein genes following IRI such as *Sparc* (Osteonectin) and *Tnc*, playing essential roles in inflammation and fibrogenesis in other organs such as the heart [71,72]. Recently, *Tnc* (protein) was also found to be important in kidney fibrosis in promoting fibroblast proliferation [73]. IR+UNx was also associated with the down-regulation of complement factor and receptor genes as well as the *Vcam1* gene, consistent with earlier findings, and probably contributing to the reduced infiltration of immune cells in our model [11,74,75].

Taken together, our results show that 1) UNx changed the transcriptional profile in the remaining non-ischemic and post-ischemic kidney, 2) only a relatively small number of genes were differentially expressed after UNx; however, in the remaining post-ischemic kidney these genes were functionally related. 3) UNx was associated with diminished structural ECM gene expression. 4) This may be mediated by inhibition of pericyte/myofibroblast activation (through reduced expression of *Pdgfra*, *Cdh11*, *Tgfb1*, and matricellular genes), and reduced macrophage infiltration in the post-ischemic kidney. Our data points to the pericyte/myofibroblast as a potential target for modulating the inflammatory response both in the early injury phase, but also later in persistent chronic inflammation and fibrogenesis. Our study is limited by having only data from one time point. Hence, we cannot firmly conclude what happens in later stages of the injury phase, the recovery phase or long-term, on the occurrence of tissue injury, inflammation and fibrosis in this model.

## Conclusion

This study provides new insights into the potential renoprotective mechanisms triggered by UNx against renal IRI. UNx reduced ischemic histopathologic injury and this could be attributed to enhanced perfusion in the inner parts of the post-ischemic kidney, and a marked attenuation of the post-ischemic pro-inflammatory and fibrogenic response by targeting the pericyte/myofibroblast. These findings could be either linked or independently occurring events, potentially protecting the kidney. The myofibroblast may have a more prominent role in the very early inflammatory and fibrogenic response following renal IRI than earlier recognised.

## Supporting information

S1 Fig The number of differentially regulated genes in response to unilateral nephrectomy and/or ischemia-reperfusion injury. The bars represent the number of significantly regulated genes, both up- and down-regulated genes. The specified groups were compared using unpaired two-class Significance Analysis of Microarrays at a false discovery rate (FDR) of 5 (white bars) or 10 (grey bars) %. A vast number of genes were affected by ischemia-reperfusion injury. A FDR of 10% was selected for further analyses. Number of animals, n = 3 in each group. IR, ischemia-reperfusion; UNx, unilateral nephrectomy.
(TIFF)

S2 Fig Pathway analysis. A: Enriched canonical pathways identified using Ingenuity ® Pathway Analysis. The input for the analysis was the list of differentially regulated genes down-regulated in the IR+UNx group compared with the IR group identified by Significance Analysis of Microarrays. The p-value is calculated by Fisher’s Exact Test. The null hypothesis tested is whether the molecules in the dataset participate in a function solely due to chance. A-value of < 0.05 (> 1.3 after -log10 transformation) is considered statistical significant. The bar presents the -log(p-value). The ratio is between the genes identified in the analysis out of the total number of genes in the given pathway. The calculated z-score predicts inhibition or activation of a given pathway. The absolute z-score is not given, but blue bar indicates z-score < 2 and red bar indicates z-score > 2. When z-score is between these extremes, the bar is grey or white (z-score = 0). B: The most significantly regulated network of molecules, down-regulated in response to unilateral nephrectomy. This was assigned a score of 38 out of 50 possible. The higher score, the more relationships among molecules. Direct (full-line) or indirect (dotted-line) relationship between genes. The purple color indicates that the molecule is represented among the genes in our dataset. White molecules are added from the IPA Knowledge Database. 16 molecules from the dataset are represented in this network. Number of animals, n = 3 in each group.
(TIF)

S1 Table Sequences of the primers used for QPCR. Sequences of the primers used for QPCR for measuring mRNA expressions. QPCR, quantitative polymerase chain reaction; *Col1a1*, collagen type 1 alpha 1 chain; *α-Sma*, α-smooth muscle actin; *Fn1*, fibronectin-1; *Cdh11*, cadherin-11; *Ccr2*, C-C chemokine receptor type 2; *Csf1r*, colony stimulating factor 1 receptor; *Mpeg1*, macrophage expressed 1; *Cd36*, CD36 molecule; *Pdgfra* and *-b*, platelet derived growth factor receptor alpha and beta; *Mcp1*, monocyte chemoattractant protein-1; *Vcam1*, vascular cell adhesion molecule-1; *Thbs1*, thrombospondin-1; *Tgfb1*, transforming growth factor-β1; *Gapdh*, glyceraldehyde-3-phosphate dehydrogenase.
(DOCX)

S2 Table Selected enriched functional categories, gene symbols and names. The functional categories displayed are biological processes according to the highest level of Gene Ontology annotations, defined by the Gene Ontology Consortium. http://www.geneontology.org/.
(DOCX)

S3 Table Top differentially regulated genes in response to UNx in the post-ischemic kidney. All genes were down-regulated in the comparison between the IR+UNx and IR groups. The fold change is the difference in log2 expression values. The q-value is the false discovery rate (FDR) individually calculated for each gene and may therefore surpass the FDR calculated for the entire gene set (5%). UNx, Unilateral nephrectomy; IR, ischemia-reperfusion. Number of animals, n = 3 in each group.
(DOCX)

S1 Text The national nentre for the replacement, refinement and reduction of animals in Research animal Research: Reporting in vivo experiments guidelines checklist. Including references to where the information is found within the manuscript.
(PDF)
