# Supplementary figures and images for: Unilateral nephrectomy diminishes ischemic acute kidney injury through enhanced perfusion and reduced pro-inflammatory and pro-fibrotic responses
Source: PLoS One. 2017 Dec 21;12(12):e0190009. doi: 10.1371/journal.pone.0190009 (PMC5739457; doi:10.1371/journal.pone.0190009)

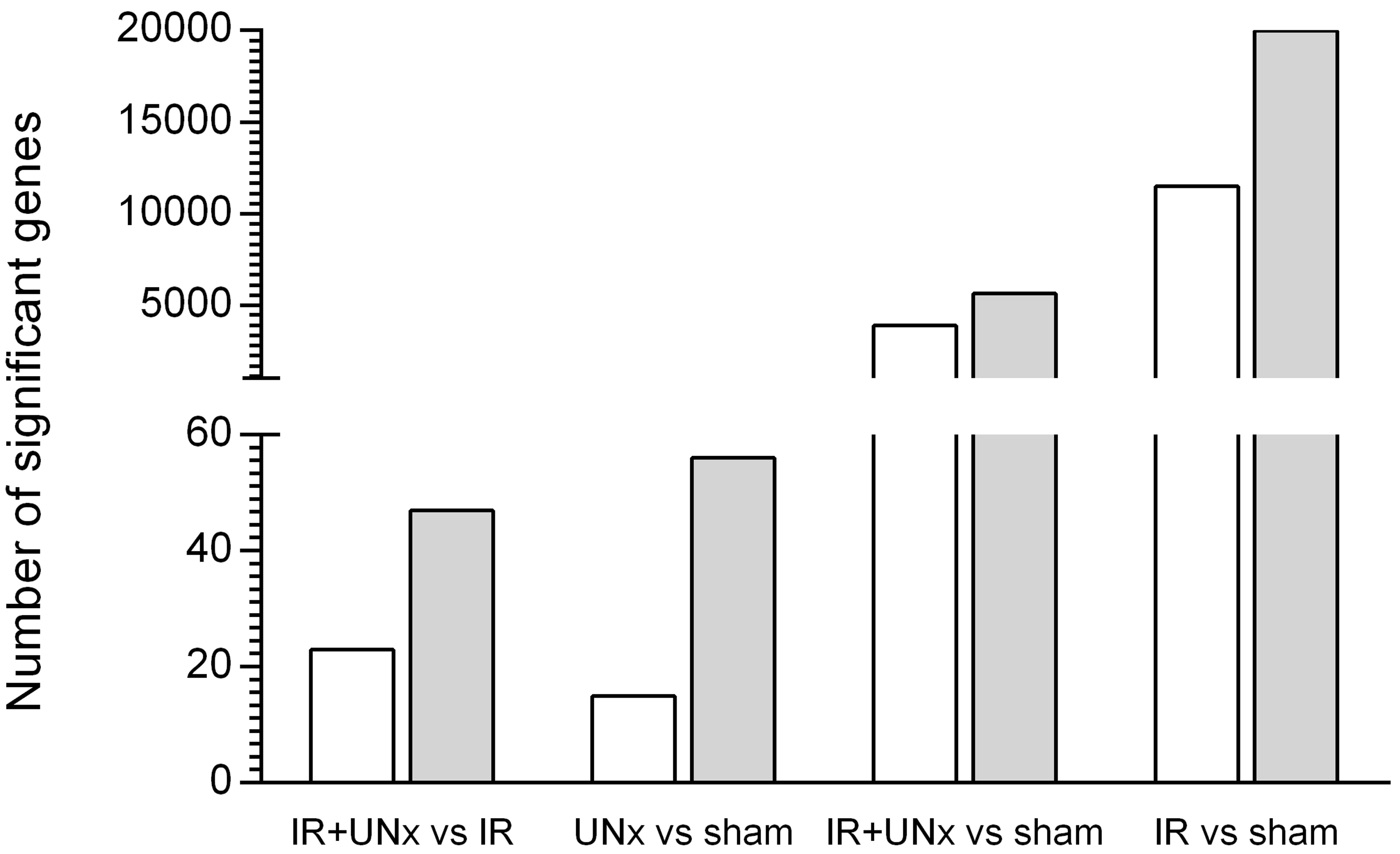

Supplement: S1 Fig — The bars represent the number of significantly regulated genes, both up- and down-regulated genes. The specified groups were compared using unpaired two-class Significance Analysis of Microarrays at a false discovery rate (FDR) of 5 (white bars) or 10 (grey bars) %. A vast number of genes were affected by ischemia-reperfusion injury. A FDR of 10% was selected for further analyses. Number of animals, n = 3 in each group. IR, ischemia-reperfusion; UNx, unilateral nephrectomy. (TIFF) [file pone.0190009.s001.tiff]

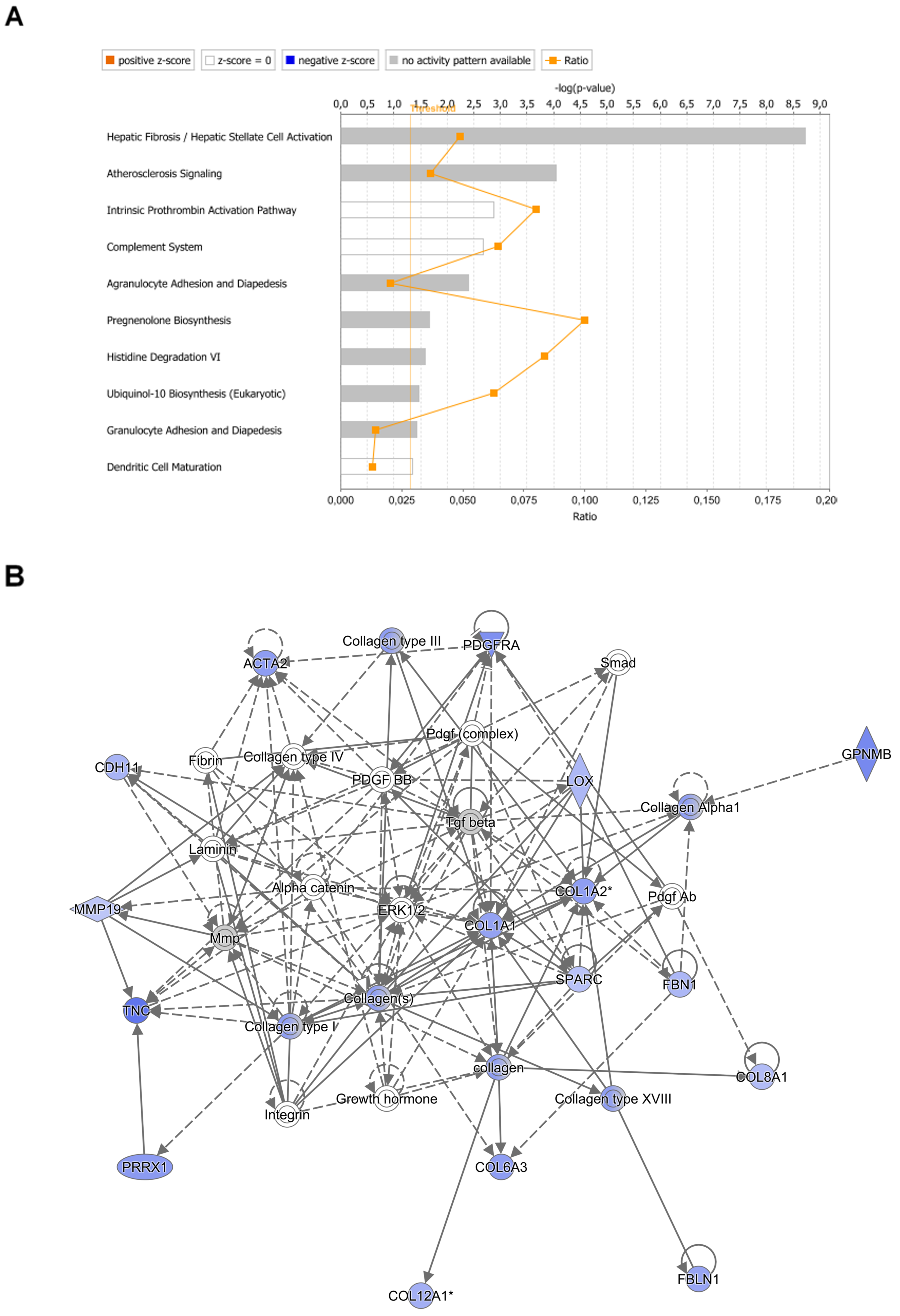

Supplement: S2 Fig — A: Enriched canonical pathways identified using Ingenuity ® Pathway Analysis. The input for the analysis was the list of differentially regulated genes down-regulated in the IR+UNx group compared with the IR group identified by Significance Analysis of Microarrays. The p-value is calculated by Fisher’s Exact Test. The null hypothesis tested is whether the molecules in the dataset participate in a function solely due to chance. A-value of < 0.05 (> 1.3 after -log10 transformation) is considered statistical significant. The bar presents the -log(p-value). The ratio is between the genes identified in the analysis out of the total number of genes in the given pathway. The calculated z-score predicts inhibition or activation of a given pathway. The absolute z-score is not given, but blue bar indicates z-score < 2 and red bar indicates z-score > 2. When z-score is between these extremes, the bar is grey or white (z-score = 0). B: The most significantly regulated network of molecules, down-regulated in response to unilateral nephrectomy. This was assigned a score of 38 out of 50 possible. The higher score, the more relationships among molecules. Direct (full-line) or indirect (dotted-line) relationship between genes. The purple color indicates that the molecule is represented among the genes in our dataset. White molecules are added from the IPA Knowledge Database. 16 molecules from the dataset are represented in this network. Number of animals, n = 3 in each group. (TIF) [file pone.0190009.s002.tif]
