# Supplementary material for: Unilateral nephrectomy diminishes ischemic acute kidney injury through enhanced perfusion and reduced pro-inflammatory and pro-fibrotic responses
Source: PLoS One. 2017 Dec 21;12(12):e0190009. doi: 10.1371/journal.pone.0190009 (PMC5739457; doi:10.1371/journal.pone.0190009)
Supplement: S1 Table — Sequences of the primers used for QPCR for measuring mRNA expressions. QPCR, quantitative polymerase chain reaction; Col1a1, collagen type 1 alpha 1 chain; α-Sma, α-smooth muscle actin; Fn1, fibronectin-1; Cdh11, cadherin-11; Ccr2, C-C chemokine receptor type 2; Csf1r, colony stimulating factor 1 receptor; Mpeg1, macrophage expressed 1; Cd36, CD36 molecule; Pdgfra and -b, platelet derived growth factor receptor alpha and beta; Mcp1, monocyte chemoattractant protein-1; Vcam1, vascular cell adhesion molecule-1; Thbs1, thrombospondin-1; Tgfb1, transforming growth factor-β1; Gapdh, glyceraldehyde-3-phosphate dehydrogenase. (DOCX) [file pone.0190009.s003.docx]

| **Primer** | **Sequence** |
| --- | --- |
| *Col1a1* | *sense 5’TCA AGA TGG TGG CCG TTA CT’3*  *antisense 5’CAT CTT GAG GTC ACG GCA TG’3* |
| *α-Sma* | *sense 5’CAT CAT GCG TCT GGA CTT GG’3*  *antisense 5’CCA GGG AAG AAG AGG AAG CA’3* |
| *Fn1* | *sense 5’CCG AAT CAC AGT AGT TGC GG’3*  *antisense 5’GCA TAG TGT CCG GAC CGA TA’3* |
| *Cdh11* | *sense 5’GGA CTC TCA GGG ACA ACC AA‘3*  *antisense 5’AGG CTT TTT CAG CTT CAC CA‘3* |
| *Ccr2* | *sense 5’CTG CCC CTA CTT GTC ATG GT‘3*  *antisense 5’AAC GCA GCA GTG TGT CAT TC‘3* |
| *Csf1r* | *sense 5’CCC TAG GAC AAA GCA AGC AG‘3*  *antisense 5’TTG CCC TCG TAG CTC TCA AT‘3* |
| *Mpeg1* | *sense 5’ATG ATT CGT GTG ATG CGA AA‘3*  *antisense 5’TGG GTC TGA GAC AGC AGA TG‘3* |
| *Cd36* | *sense 5’CTCTGACATTTGCAGGTCCA‘3*  *antisense 5’CAGGTCTTTCCTTCTTTGGCAC‘3* |
| *Pdgfra* | *sense 5’ACGTTCAAGACCAGCGAGTT‘3*  *antisense 5’CAGTTTGATGGACGGGAGTT‘3* |
| *Pdgfrb* | *sense 5’TGTTCGTGCTATTGCTCCTG‘3*  *antisense 5’TCAGCACACTGGAGAAGGTG‘3* |
| *Mcp1* | *sense 5’TAG CAT CCA CGT GCT GTC TC‘3*  *antisense 5’CCG ACT CAT TGG GAT CAT CT‘3* |
| *Vcam1* | *sense 5’CGG TCA TGG TCA AGT GTT TG‘3*  *antisense 5’TTG GGG GAG GTG TAG ACT TG‘3* |
| *Thbs1* | *sense 5’TGT GAC AAT TGC CCC TAC AA‘3*  *antisense 5’GTG TCC CTC TGA TCC ACG TT‘3* |
| *Tgfb1* | *sense 5’TGA GTG GCT GTC TTT TGA CG3’*  *antisense 5’TTC TCT GTG GAG CTG AAG CA’3* |
| *Gapdh* | *sense 5’TAA AGG GCA TCC TGG GCT ACA CT‘3*  *antisense 5’TTA CTC CTT GGA GGC CAT GTA GG‘3* |
