# Supplementary material for: Unilateral nephrectomy diminishes ischemic acute kidney injury through enhanced perfusion and reduced pro-inflammatory and pro-fibrotic responses
Source: PLoS One. 2017 Dec 21;12(12):e0190009. doi: 10.1371/journal.pone.0190009 (PMC5739457; doi:10.1371/journal.pone.0190009)
Supplement: S2 Table — The functional categories displayed are biological processes according to the highest level of Gene Ontology annotations, defined by the Gene Ontology Consortium. http://www.geneontology.org/. (DOCX) [file pone.0190009.s004.docx]

| **GO annotation** | **Gene** | **Name** |
| --- | --- | --- |
| Wound healing | Cnn2 | Calponin 2 |
|  | Col1a1 | Collagen type 1 alpha 1 chain |
|  | Fn1 | Fibronectin 1 |
|  | Lox | Lysyl oxidase |
|  | Pdgfra | Platelet derived growth factor receptor alpha |
|  | Sparc | Secreted protein acidic and cysteine rich |
|  | Tnc | Tenascin C |
| ECM organization | Abi3bp | ABI family member 3 binding protein |
|  | Col1a1 | - |
|  | Col1a2 | Collagen type I alpha 2 chain |
|  | Fn1 | *-* |
|  | Fbln1 | Fibulin 1 |
|  | Pdgfra | - |
| Cell adhesion | Col12a1 | Collagen type XII alpha 1 chain |
|  | Fn1 | - |
|  | Gpnmb | Glycoprotein nmb |
|  | Lsamp | Limbic system-associated membrane protein |
|  | Tnc | *-* |
|  | Vcam1 | Vascular cell adhesion molecule 1 |
| Positive regulation of fibroblast proliferation | Fn1 | - |
|  | Fbln1 | - |
|  | Pdgfra | - |
| Immune response | Cd36 | CD36 molecule |
|  | Colec12 | Collectin sub-family member 12 |
|  | C1gb | Complement C1q B chain |
|  | C7 | Complement C7 |
