# Supplementary material for: Unilateral nephrectomy diminishes ischemic acute kidney injury through enhanced perfusion and reduced pro-inflammatory and pro-fibrotic responses
Source: PLoS One. 2017 Dec 21;12(12):e0190009. doi: 10.1371/journal.pone.0190009 (PMC5739457; doi:10.1371/journal.pone.0190009)
Supplement: S3 Table — All genes were down-regulated in the comparison between the IR+UNx and IR groups. The fold change is the difference in log2 expression values. The q-value is the false discovery rate (FDR) individually calculated for each gene and may therefore surpass the FDR calculated for the entire gene set (5%). UNx, Unilateral nephrectomy; IR, ischemia-reperfusion. Number of animals, n = 3 in each group. (DOCX) [file pone.0190009.s005.docx]

| *Gene Symbol* | *Name* | *Entrez ID* | *UniGene Accession* | *Fold change* | *q-value (%)* |
| --- | --- | --- | --- | --- | --- |
| Col1a2 | collagen type I alpha 2 chain | 84352 | ENSRNOT00000016423 | -2.74 | 0.00 |
| Col1a2 | collagen type I alpha 2 chain | 84352 | NM_053356 | -2.63 | 0.00 |
| Fbln1 | fibulin 1 | 315191 | NM_001127547 | -2.56 | 0.00 |
| Rcn3 | reticulocalbin | 494125 | NM_001008694 | -2.01 | 0.00 |
| Lox | lysyl oxidase | 24914 | NM_017061 | -2.00 | 0.00 |
| Cthrc1 | collagen triple helix repeat containing 1 | 282836 | NM_001271300 | -2.00 | 0.00 |
| Col12a1 | collagen type XII alpha 1 chain | 25683 | ENSRNOT00000043691 | -2.25 | 0.00 |
| sluzaw |  |  | Unannotated AceView Transcript | -1.52 | 0.00 |
| Lsamp | limbic system-associated membrane protein | 29561 | NM_017242; BC087607 | -1.51 | 0.00 |
| Tagln | transgelin | 25123 | NM_031549 | -2.41 | 4.57 |
| Sparc | secreted protein acidic and cysteine rich | 24791 | NM_012656 | -1.71 | 3.81 |
| Prcp | prolylcarboxypeptidase | 293118 | NM_001106281 | -1.94 | 3.81 |
| Cd36 | cd36 molecule | 29184 | NM_031561 | -1.69 | 7.04 |
| Prrx1 | paired related homeobox 1 | 266813 | NM_153821 | -2.85 | 6.53 |
| Tnc | tenascin C | 116640 | NM_053861 | -3.94 | 6.10 |
| Pdgfra | platelet derived growth factor receptor alpha | 25267 | NM_012802 | -3.13 | 5.08 |
| Olfml3 | olfactomedin-like 3 | 310743 | NM_001107708 | -1.75 | 5.08 |
| sawla |  |  | Unannotated AceView Transcript | -2.28 | 5.08 |
| Acta2 | actin, alpha 2, smooth muscle, aorta | 81633 | NM_031004 | -2.76 | 4.81 |
| KnowTID_00008038 |  |  | Unannotated AceView Transcript | -1.58 | 4.57 |
| Gpnmb | glycoprotein nmb | 113955 | NM_133298 | -3.33 | 4.16 |
| Cybrd1 | cytochrome b reductase 1 | 295669 | NM_001011954 | -1.75 | 4.16 |
| Fn1 | fibronectin | 25661 | NM_019143 | -2.95 | 3.98 |
|  | | | | | |
